# Estimation of the Harvey Bradshaw Index from the Patient-Reported Outcome 2 in Crohn’s Disease: Results Based on a Large Scale Randomized Controlled Trial

**DOI:** 10.1093/ibd/izae281

**Published:** 2024-12-04

**Authors:** Reena Khanna, Surim Son, Guangyong Zou, Pavel S Roshanov

**Affiliations:** Department of Medicine, Division of Gastroenterology, Western University, London, Ontario, Canada; Department of Epidemiology and Biostatistics, Western University, London, Ontario, Canada; Lawson Health Research Institute, London, Ontario, Canada; Department of Epidemiology and Biostatistics, Western University, London, Ontario, Canada; Department of Epidemiology and Biostatistics, Western University, London, Ontario, Canada; Alimentiv Inc., London, Ontario, Canada; Department of Epidemiology and Biostatistics, Western University, London, Ontario, Canada; Lawson Health Research Institute, London, Ontario, Canada; Department of Medicine, Division of Nephrology, Western University, London, Ontario, Canada; Population Health Research Institute, Hamilton, Ontario, Canada

**Keywords:** Crohn’s disease, correlation, Harvey–Bradshaw Index, patient-reported outcome, symptom scores

## Abstract

**Background:**

Many registrational trials in Crohn’s disease assess treatment efficacy with the 2-item Patient-Reported Outcome (PRO2), while the Harvey–Bradshaw Index (HBI) is prominent in pragmatic trials and clinical practice. The translation between PRO2 and HBI has not been established.

**Methods:**

Data from a Phase 3 trial of vedolizumab in Crohn’s disease were used to determine the Pearson correlation between PRO2 and HBI. Linear regression was used to fit equations that estimate between indices; 95% prediction intervals were determined for HBI scores corresponding to PRO2 thresholds for disease activity. Internal validation of the conversion equations was performed using the bootstrap methods.

**Results:**

PRO2 and HBI were highly correlated at baseline (*r* = 0.75 95% confidence interval (CI) 0.73-0.78; *P* < .001), induction (*r* = 0.87; 95% CI, 0.85-0.88; *P* < .001), and maintenance (*r* = 0.88; 95% CI, 0.85-0.90; *P* < .001). PRO2 and HBI change scores were moderately correlated (*r* = 0.72; 95% CI 0.69-0.75; *P* < .001) in induction and more strongly correlated during maintenance (*r* = 0.81; 95% CI 0.78- 0.84; *P* < .001). Regression equations for conversion of PRO2 to HBI from all cohorts (induction, maintenance, randomized, open-label) support an approximate conversion where HBI = 0.5 PRO2. As expected from the imperfect correlation between scores, the prediction intervals were generally wide. No evidence of overfitting was seen in bootstrap internal validation.

**Conclusions:**

PRO2 and HBI correlate strongly and conversion between them is possible. These findings facilitate the practical application of trial results and clinical guidelines.

**Key Messages**

- Conventionally, the Harvey–Bradshaw Index has been used in clinical practice whereas the PRO2 has been used in clinical trials.
- This article provides an equation for estimation between the two indices.
- These data are important to interpret clinical results and to apply results from trials to patient care.

## Introduction

The Crohn’s Disease Activity Index (CDAI) and the 2-item patient-reported outcome (PRO2) have been widely used for symptom assessment in clinical trials of Crohn’s disease. In contrast, the Harvey-Bradshaw Index (HBI) has been used in pragmatic trials and clinical practice.

The CDAI served as the primary outcome measure for the regulatory approval of drug therapies for several decades. This index is a composite outcome which consists of 8 items including symptoms, signs, and laboratory parameters.^1,2^ The CDAI has been criticized for several reasons. First, the patient-reported items provide most of the “signal,” whereas the remaining items contribute to measurement noise.^3,4^ Second, the index correlates poorly with active inflammation.^5^ Third, since only 3 items––number of bowel movements, abdominal pain, and general well-being––are provided by patients, many regulatory bodies do not classify CDAI as a patient-reported outcome.

Regulators, such as the European Medicines Agency and Health Canada, now mandate that PROs are incorporated into the primary outcome measure for trials in Crohn’s disease.^6^ PRO2 was developed from the patient diary card items of the CDAI for use in clinical trials and assesses stool frequency and abdominal pain.^6^

In contrast, the HBI^7^ has conventionally been used in clinical practice and pragmatic trials^8^ because it is intuitive and does not require laboratory investigations. The score assesses abdominal pain, stool frequency, general well-being, presence of abdominal masses, and extra-intestinal manifestations of IBD.

It is unclear how to estimate PRO2 from HBI in practice. Translation is needed when counseling patients regarding the degree of benefit they may expect with a proposed therapy, in applications to cover medication costs in some jurisdictions, and in the clinical interpretation of trial results. We used data from a phase 3 clinical trial of a treatment of known efficacy in Crohn’s disease to address this need.

## Materials and Methods

### Study Design

This was a secondary analysis of data from GEMINI 2,^9^ a phase 3 parallel-group, double-blinded, randomized control trial of vedolizumab in Crohn’s disease. The induction trial of GEMINI 2 consisted of 2 cohorts: (1) 368 patients with moderate-to-severely active Crohn’s disease were randomized to receive vedolizumab or placebo at Week 0 and 2 weeks; (2) Seven hundred and forty-seven patients received open-label vedolizumab at the same time points. At Week 6, 461 patients who demonstrated response to vedolizumab—defined as a decrease in CDAI ≥70 points—were randomized 1:1:1 to receive vedolizumab every 8 weeks, vedolizumab every 4 weeks, or placebo every 8 weeks until Week 52 in the maintenance trial. Data were obtained from a publicly accessible platform, Vivli (https://vivli.org).

### Patients

Eligible patients for GEMINI 2 were 18–80 years old; with a diagnosis of Crohn’s disease for a minimum of 3 months; a baseline CDAI score of 220-450; and one objective marker of disease activity including a C-reactive protein >2.87 mg L^−1^, colonoscopy demonstrating ≥3 large ulcers or ≥10 aphthous ulcers, or a fecal calprotectin >250 µg per gram of stool with radiographic evidence of ulcers.

### Clinical Assessments

Clinical trial assessments were performed at weeks 0, 2, 4, and 6 during the induction trial, and every 4 weeks during the maintenance trial until Week 52.

### Crohn’s Disease Activity Index/2-Item Patient-Reported Outcome

The Crohn’s Disease Activity Index^1,2^ was assessed at screening, Week 0, 2, 4, and 6, which comprised the induction period, and then every 4 weeks thereafter until Week 52 in the maintenance period. The diary questions from the CDAI were used to calculate PRO2 as the sum of 7-day average of liquid stools and abdominal pain, weighted using the CDAI multiplication factors. CDAI scores range from 0 to 600 with higher scores indicating greater disease activity. Widely used benchmarks are CDAI <150 clinical remission, 150-219 mildly active disease, 220-450 moderately active disease, and above 450 severe disease. PRO2 scores are heavily dependent on patient-reported stool frequency, with higher scores indicating greater symptom severity. Based on correlation to CDAI thresholds, a total PRO2 score of <8 represents clinical remission, 8-14 suggests mildly active disease, and 14-34 indicates moderately active disease, and >34 represents severely active disease. Similarly, widely used thresholds for CDAI response of a decrease in 50, 70, or 100 points correlated to PRO2 changes of 2, 5, and 8-points from baseline.^10^

### HBI

Harvey-Bradshaw Index^7^ was assessed at screening, Week 0, 2, 4, and 6, and every 4 weeks thereafter until week 52. Harvey-Bradshaw Index consists of 5 domains, namely abdominal mass, abdominal pain, complications, general well-being, and the number of liquid or very soft stools. Scores from all 5 domains were summed to calculate the total HBI score. The total HBI scores can range from 0 to >16, depending on the number of daily stools reported by patients. Based on convention, a total HBI score of <5 indicates remission, 5-7 suggests mild severity, 8-16 denotes moderate severity, and ≥16 demarks severe disease.

Harvey-Bradshaw Index and PRO2 were estimated in 3 cohorts: (1) overall (randomized and open-label cohorts); (2) randomized population; and (3) open-label cohort. To determine the effect of blinding on the relationship between PRO2 and HBI, an interaction term was added between PRO2 and a variable that denoted randomized versus open-label cohort. If the interaction with blinding did not add significant information to the equation without this variable, then the simple equation was considered sufficient.

### Data Analysis

Patient characteristics were summarized using mean and SD for continuous variables, and frequency and percentages for categorical variables.

Data collected during the induction and maintenance phases were analyzed separately to determine thresholds for HBI in populations with moderate-to severely active disease and mild to moderately active disease, respectively. In the induction phase, clinical remission was assessed using the total PRO2 and HBI scores obtained at Week 6, whereas clinical response was assessed using change scores from baseline to Week 6. Corresponding analyses were performed at Week 52 for the maintenance phase. The relationship between the HBI and PRO2 scores was assessed with Pearson correlation. Linear regression was used to estimate PRO2 score from HBI scores, and to identify corresponding HBI scores for established PRO2 thresholds for disease severity. Linear regression was also used to estimate HBI change scores from PRO2 change scores, and to identify HBI scores for established PRO2 thresholds for clinical response; 95% prediction intervals were calculated. Internal validation of the equations for estimation was conducted using 1000 bootstrap resamples to evaluate model performance.

Statistical analyses were performed using SAS 9.4 (SAS Institute Inc., Cary, NC, USA) and R (version 4.3, R Foundation for Statistical Computing, Vienna, Austria). GEMINI 2 was approved by research ethics boards at participating centers, and this analysis was approved by the research ethics board at Western University.

## Results

### Study Population

This study included 12 392 pairs of HBI and PRO2 measurements from 1102 patients during the induction and maintenance phases of GEMINI 2. The relevant demographic information by cohort is summarized in Table 1. Since data from all 3 cohorts were similar, only analyses for the overall population that comprises both the randomized and open-label cohorts are shown below. Data from the randomized and open-label cohorts assessed individually are available in the supplement.

**Table 1.** Baseline demographic data of patients in GEMINI 2.

	Overall (*N* = 1029)	Randomized (*n* = 346)	Open-label (*n* = 683)
Age, mean (SD)	36.3 (12.2)	37.5 (12.3)	35.7 (12.1)
Sex, *n* (%)			
Female	539 (52.4%)	179 (51.7%)	360 (52.7%)
Male	490 (47.6%)	167 (48.3%)	323 (47.3%)
Race, *n* (%)			
American	2 (0.2%)	1 (0.3%)	1 (0.1%)
Asian	84 (8.2%)	51 (14.7%)	33 (4.8%)
Black	21 (2.0%)	6 (1.7%)	15 (2.2%)
Native	1 (0.1%)	0 (0%)	1 (0.1%)
Other	4 (0.4%)	1 (0.3%)	3 (0.4%)
White	917 (89.1%)	287 (82.9%)	630 (92.2%)
Smoking status, *n* (%)			
Current smoker	279 (27.1%)	83 (24.0%)	196 (28.7%)
Former smoker	245 (23.8%)	73 (21.1%)	172 (25.2%)
Never smoked	504 (49.0%)	190 (54.9%)	314 (46.0%)
Fistula, *n* (%)			
No	849 (82.5%)	276 (79.8%)	573 (83.9%)
Yes	167 (16.2%)	62 (17.9%)	105 (15.4%)
Missing	13 (1.3%)	8 (2.3%)	5 (0.7%)
Crohn’s disease duration (years), mean (SD)	9.08 (7.88)	8.79 (8.12)	9.23 (7.75)
Disease localization, *n* (%)			
Colon only	287 (27.9%)	101 (29.2%)	186 (27.2%)
Ileocolonic	568 (55.2%)	187 (54.0%)	381 (55.8%)
Ileum only	174 (16.9%)	58 (16.8%)	116 (17.0%)
Baseline CRP (mg L^−1^), mean (SD)	20.1 (25.4)	21.7 (24.8)	19.3 (25.7)
Baseline Fecal Calprotectin (mg kg^−1^), mean (SD)	1210 (1800)	1560 (2280)	1040 (1470)
Baseline CDAI score, mean (SD)	321 (68.1)	324 (72.9)	320 (65.6)
Baseline PRO2 score, mean (SD)	21.6 (6.72)	21.2 (6.81)	21.8 (6.67)
Baseline HBI score, mean (SD)	11.1 (3.66)	11.0 (3.71)	11.2 (3.63)

Abbreviations: CDAI, Crohn’s Disease Activity Index; HBI, Harvey–Bradshaw Index; PRO2, 2-item patient-reported outcome.

### Relationship Between HBI and PRO2

**Figure 1.**
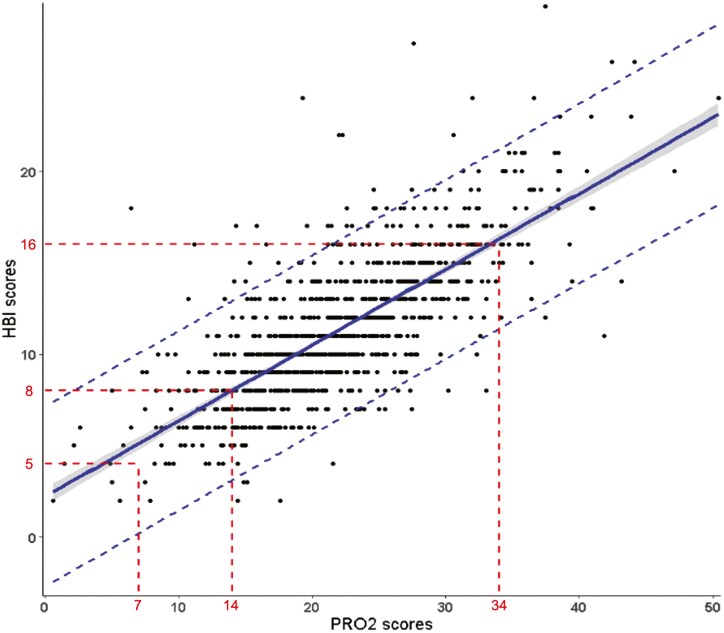
The relationship between Harvey–Bradshaw Index (HBI) and 2-item patient-reported outcome scores at Week 0. The shaded area represents the 95% confidence interval (CI). Dashed lines represents the 95% prediction interval. The 95% confidence interval represents uncertainty in the estimate of the mean response for given *X* values. The 95% prediction interval, on the other hand, provides a range within which we can expect to find individual future observations of the dependent variable (*Y*) for a given value of the independent variable (*X*). This interval is wider than the CI because it accounts for both the uncertainty in estimating the true regression line and the inherent variability in individual observations. The prediction interval is useful when making predictions for a new patient and needed to understand the range within which that prediction is likely to fall.

**Figure 2.**
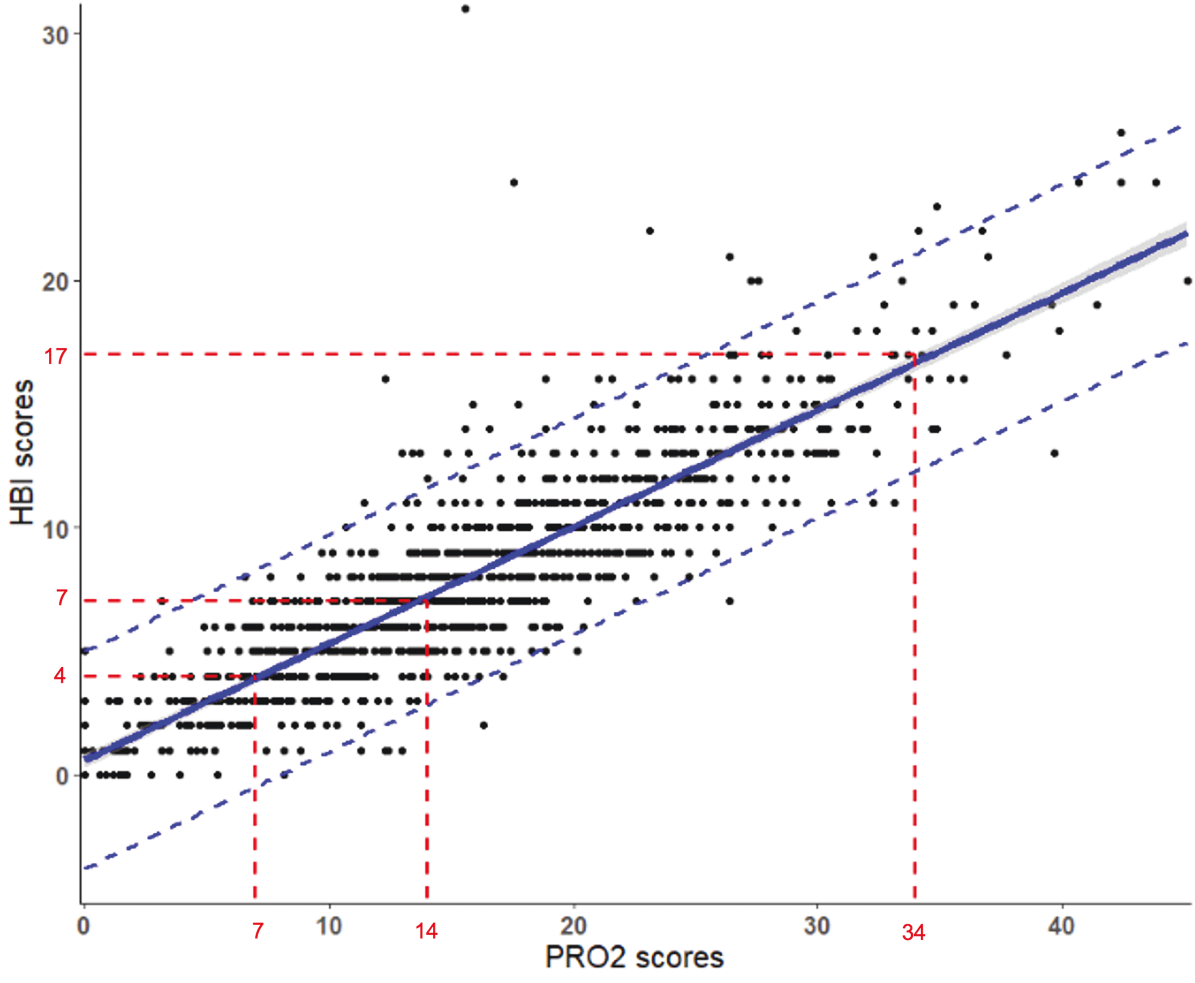
The relationship between Harvey–Bradshaw Index and PRO2 scores in the induction phase. The shaded area represents the 95% confidence interval. Dashed lines represents the 95% prediction interval.

**Figure 3.**
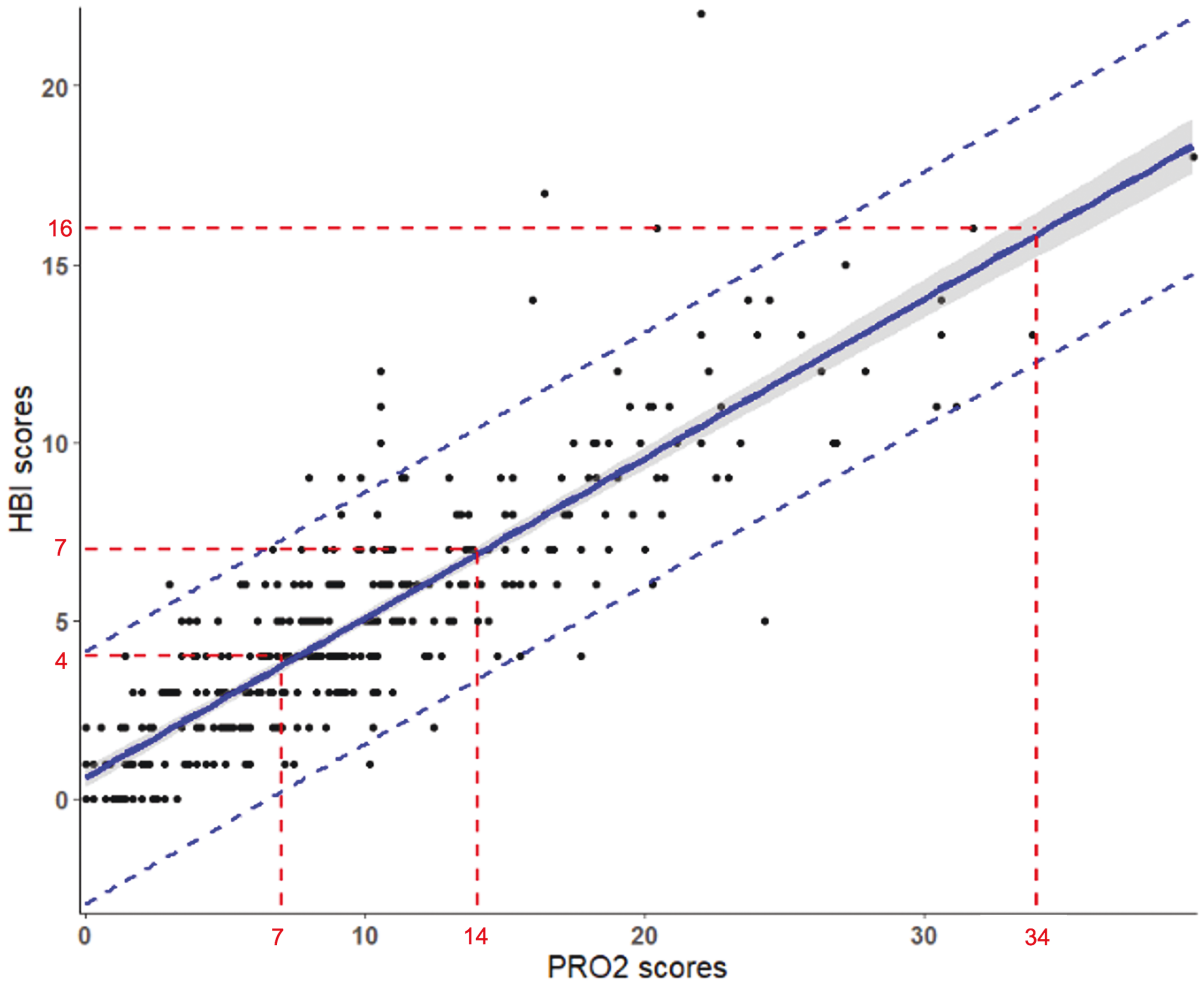
The relationship between Harvey–Bradshaw Index and PRO2 scores in the maintenance phase. The shaded area represents the 95% confidence interval. The dashed lines represents the 95% prediction interval.

2-Item patient-reported outcome scores ranged from 0 to 45 and HBI scores ranged from 0 to 31. 2-Item patient-reported outcome and HBI scores were highly correlated at baseline (Pearson *r* = 0.75 95% confidence interval (CI) 0.73-0.78; *P* < .001), in the induction (*r* = 0.87; 95% CI 0.85-0.88; *P* < .001), and maintenance phase (*r* = 0.88; 95% CI, 0.85-0.90; *P* < .001). Figures 1-3 show the relationship between HBI and PRO2 scores at baseline, and in induction and maintenance phases respectfully.

**Figure 4.**
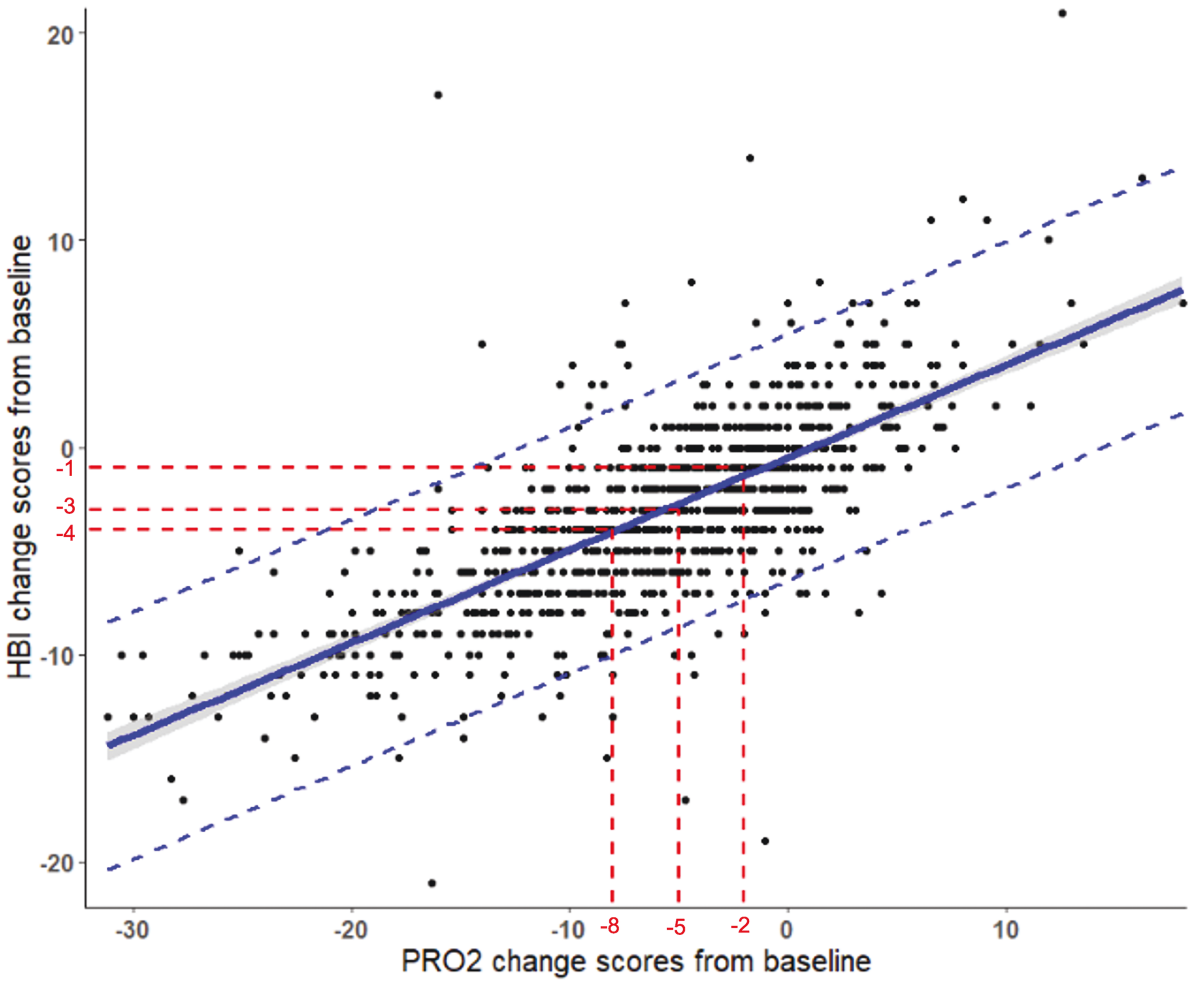
The relationship between Harvey–Bradshaw Index change scores and PRO2 change scores in the induction phase. The shaded area represents the 95% confidence interval. Dashed lines represents the 95% prediction interval.

**Figure 5.**
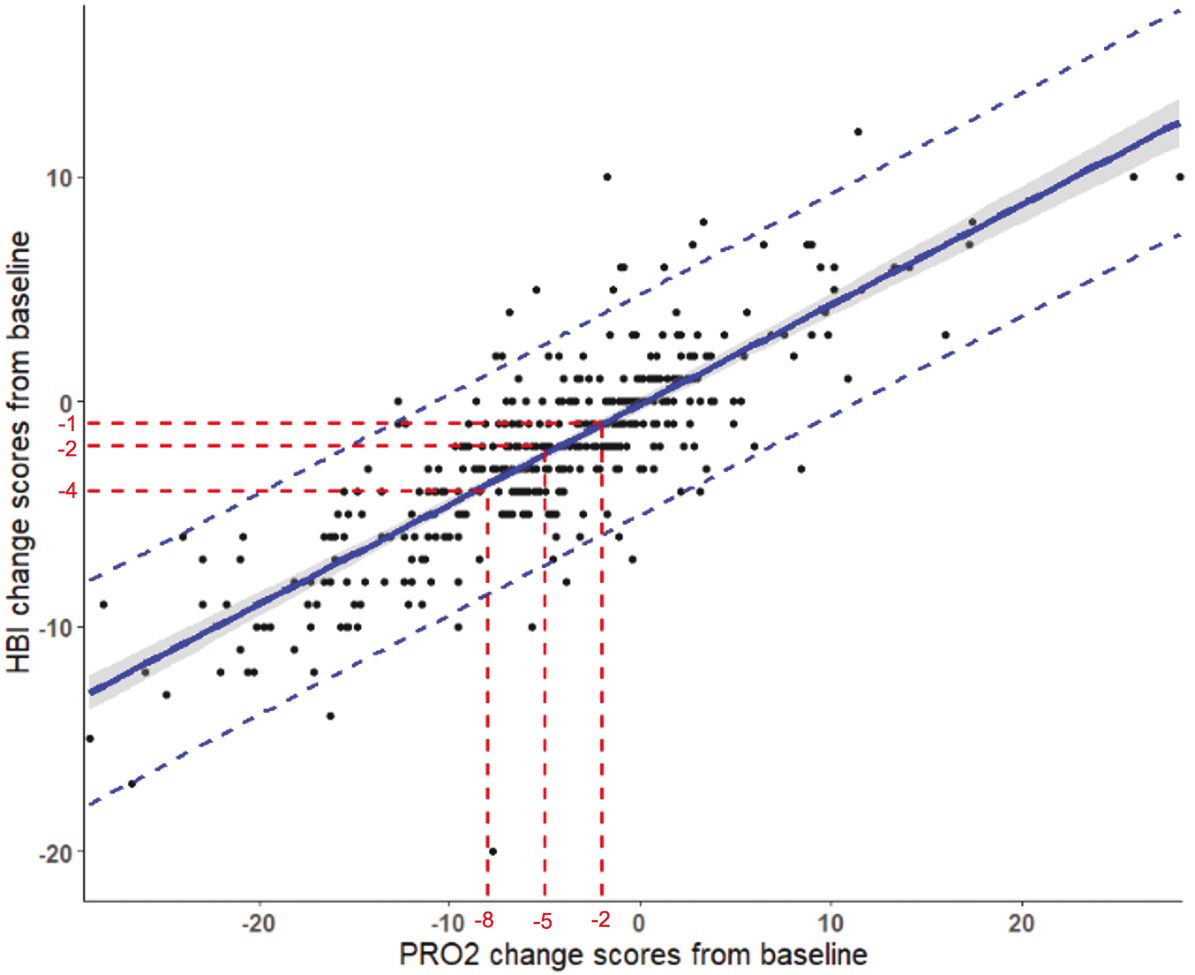
The relationship between Harvey–Bradshaw Index change scores and PRO2 change scores in the maintenance phase. Shadied areas represents the 95% confidence interval. The dashed lines represents the 95% prediction interval.

Harvey-Bradshaw Index change scores ranged from −21 to 21, and PRO2 change scores ranged from −31 to 18. 2-Item patient-reported outcome and HBI changes were moderately correlated in the induction phase (*r* = 0.72; 95% CI 0.69, 0.75; *P* < .001) and more strongly correlated in the maintenance phase (*r* = 0.81; 95% CI 0.78-0.84; *P* < .001). Figures 4 and 5 show the relationship between HBI and PRO2 change scores in the induction and maintenance phases respectfully.

The corresponding relationships for the randomized and open-label cohorts assessed individually are shown in Supplementary Table 1.

### Estimation of HBI from PRO2

Regression equations for conversion of PRO2 to HBI from all cohorts supports an equation of HBI = 0.5PRO2. Equations from each individual cohort were similar: absolute value induction phase HBI = 0.59 + 0.47PRO2 (*R*^2^ = 0.75; *P* < .001), absolute value maintenance phase HBI = 0.60 + 0.45PRO2 (*R*^2^ = 0.77; *P* < .001), change score induction phase HBI = 0.59 + 0.47PRO2 (*R*^2^ = 0.75; *P* < .001), and change score maintenance phase HBI = 0.60 + 0.45PRO2 (*R*^2^ = 0.77; *P* < .001). The *R*^2^ values suggest the equations explain most of the variation in HBI. The equation for each individual cohort is shown in Supplementary Tables 2 and 3. No evidence of overfitting was observed in bootstrap internal validation (Supplementary Tables 4 and 5, Supplementary Figures 1-5). Regression models to assess assumptions are provided in Supplementary Figure 6.

### Estimation of Key Absolute Thresholds

PRO2 scores of 8, 14, and 34 correspond to HBI score of approximately 4, 7, and 16 in both the induction and maintenance phases. These thresholds with 95% prediction intervals are shown in Table 2. Threshold scores determined from each cohort, individually, are shown in Supplementary Table 6.

**Table 2.** Harvey–Bradshaw Index scores corresponding to PRO2 score in the overall cohort by trial phase.

	Induction		Maintenance	
PRO2	HBI	95% Prediction interval	HBI	95% Prediction interval
1	1.1	(0.0,5.5)	1.1	(0.0,4.6)
2	1.5	(0.0,5.9)	1.5	(0.0,5.0)
3	2.0	(0.0,6.4)	1.9	(0.0,5.5)
4	2.5	(0.0,6.9)	2.4	(0.0,5.9)
5	3.0	(0.0,7.3)	2.8	(0.0,6.4)
6	3.4	(0.0,7.8)	3.3	(0.0,6.8)
7	3.9	(0.0,8.3)	3.7	(0.2,7.3)
8	4.4	(0.0,8.8)	4.2	(0.7,7.7)
9	4.9	(0.5,9.2)	4.6	(1.1,8.2)
10	5.3	(0.9,9.7)	5.1	(1.6,8.6)
11	5.8	(1.4,10.2)	5.5	(2.0,9.1)
12	6.3	(1.9,10.7)	6.0	(2.4,9.5)
13	6.7	(2.4,11.1)	6.4	(2.9,10.0)
14	7.2	(2.8,11.6)	6.9	(3.3,10.4)
15	7.7	(3.3,12.1)	7.3	(3.8,10.9)
16	8.2	(3.8,12.5)	7.8	(4.2,11.3)
17	8.6	(4.3,13.0)	8.2	(4.7,11.8)
18	9.1	(4.7,13.5)	8.7	(5.1,12.2)
19	9.6	(5.2,14.0)	9.1	(5.6,12.7)
20	10.1	(5.7,14.4)	9.6	(6.0,13.1)
21	10.5	(6.1,14.9)	10.0	(6.5,13.6)
22	11.0	(6.6,15.4)	10.5	(6.9,14.0)
23	11.5	(7.1,15.9)	10.9	(7.4,14.5)
24	11.9	(7.6,16.3)	11.4	(7.8,14.9)
25	12.4	(8.0,16.8)	11.8	(8.2,15.4)
26	12.9	(8.5,17.3)	12.2	(8.7,15.8)
27	13.4	(9.0,17.7)	12.7	(9.1,16.3)
28	13.8	(9.4,18.2)	13.1	(9.6,16.7)
29	14.3	(9.9,18.7)	13.6	(10.0,17.2)
30	14.8	(10.4,19.2)	14.0	(10.5,17.6)
31	15.2	(10.9,19.6)	14.5	(10.9,18.1)
32	15.7	(11.3,20.1)	14.9	(11.4,18.5)
33	16.2	(11.8,20.6)	15.4	(11.8,19.0)
34	16.7	(12.3,21.1)	15.8	(12.2,19.4)
35	17.1	(12.7,21.5)	16.3	(12.7,19.9)
36	17.6	(13.2,22.0)	16.7	(13.1,20.3)
37	18.1	(13.7,22.5)	17.2	(13.6,20.8)
38	18.6	(14.2,23.0)	17.6	(14.0,21.2)
39	14.8	(10.4,19.2)	14.0	(10.5,17.6)
40	19.5	(15.1,23.9)	18.5	(14.9,22.1)

Highlighted values represent established thresholds for PRO2.

Abbreviations: HBI, Harvey–Bradshaw Index; PRO2, 2-item patient-reported outcome.

### Estimation of Change Score Thresholds of HBI from PRO2

2-Item patient-reported outcome response scores of 2-points, 5-points, and 8-points correspond to HBI change score of approximately 1, 3, and 4 in the induction phase and 1, 2, and 4 in the maintenance phase. These thresholds with 95% prediction intervals are shown in Table 3. The HBI threshold scores were similar for each individual cohort and is shown in Supplementary Table 7.

**Table 3.** Harvey–Bradshaw Index (HBI) change scores corresponding to PRO2 change scores in the overall cohort by trial phase.

	Induction		Maintenance	
PRO2	HBI	95% Prediction interval	HBI	95% Prediction interval
−1	−0.9	(0.0,5.0)	−0.6	(0.0,4.3)
−2	−1.4	(0.0,4.6)	−1.0	(0.0,3.9)
−3	−1.8	(0.0,4.1)	−1.5	(0.0,3.5)
−4	−2.3	(0.0,3.7)	−1.9	(0.0,3.7)
−5	−2.7	(0.0,3.2)	−2.3	(0.0,2.6)
−6	−3.2	(0.0,2.8)	−2.8	(0.0,2.1)
−7	−3.6	(0.0,2.3)	−3.2	(0.0,1.7)
−8	−4.1	(0.0,1.9)	−3.7	(0.0,1.2)
−9	−4.5	(0.0,1.4)	−4.1	(0.0,0.8)
−10	−5.0	(0.0,1.0)	−4.6	(0.0,0.3)

Highlighted values represent established thresholds for PRO2.

Abbreviations: HBI, Harvey–Bradshaw Index; PRO2, 2-item patient-reported outcome.

## Discussion

The results of this study demonstrate that PRO2 and HBI correlate strongly. Moreover, regression analysis identified a single equation for estimation between these indices for both the induction and maintenance phases. Our results suggest a estimation of 2:1 for PRO2:HBI. High-performing thresholds for HBI that translate to established PRO2 thresholds and change scores have been determined and support this 2:1 ratio. Notably no difference was noted among the overall, randomized, and open-label cohorts.

Thresholds have been established in both a population with moderate-to-severely active disease using data from the induction phase and in those with response to therapy in the maintenance phase since disease activity was previously shown to influence thresholds for PRO2.^11^ PRO2 and HBI are most likely to be applied to patients with active disease who receive therapy to assess for treatment success. The population in the current analysis approximates this target group and provides relevant data. It also provides thresholds to monitor patients following response to therapy.

Notably the *R*^2^ for the equations were not robust, likely due to limitations from noise in the indices. In particular, HBI is encumbered by a subjective element—general well-being—which may be influenced by factors that are unrelated to disease activity such as mood or social functioning. These factors may not be directly responsive to therapy which adds “noise” to the measurement index. Furthermore, unlike PRO2, HBI accounts for complications of disease. Although the majority of most of these manifestations may respond to therapy, other items—such as an abscess—would not respond to immunosuppressive therapies. In this analysis, PRO2 was calculated using the mean score for abdominal pain and stool frequency over 7 days and weighted by the CDAI multiplication factor^10^; however, HBI is typically calculated using data from 1 day prior to the assessment.^7^ Given the daily fluctuation in symptoms, an averaged score would likely provide more accurate assessments of symptoms. However, documenting stool frequency for a week can be cumbersome for patients, conventionally resulting in HBI to be preferred in many clinical settings. As a result, PRO2 may be more robust for use in clinical trials and in clinical practice to determine treatment success. However, additional aspects of disease that influence well-being such as complications require an alternate index.

Moreover, the wide prediction intervals likely reflect the imperfect correlation between HBI and PRO2. The prediction interval provides the range values for HBI that can be expected for new patients with the same PRO2 score. It accounts for variability in estimation of the regression line and in individual observations. The wide intervals suggest a score consistent with remission may not accurately predict this disease state. This finding is consistent with clinical guidelines that advise use of clinical indices in combination with objective markers,^12^ due to the recognized poor operating properties of the clinical indices.

There are limitations to this work. First, data were included from a single Phase 3 study. However, few studies have captured both PRO2 items and HBI. GEMINI 2^9^ included a large sample allowing for robust analysis. Second, data for PRO2 were calculated from CDAI. PRO2 was developed from CDAI, and this derivation has been well studied.^4^ However, only one definition of PRO2 was assessed in this analysis. Due to the multitude of definitions, the original PRO2^10^ calculation was chosen since it has been correlated to both CDAI and the widely used thresholds for disease activity and clinical response. Third, the estimation equations were not externally validated but they are expected to perform well in new patients for 2 reasons: (1) the equations were nearly identical across all cohorts and time points in this study; (2) no evidence of overfitting was observed in bootstrap internal validation. Finally, we are uncertain whether the prediction interval for the estimation coefficient between HBI and PRO2 are sufficiently reliable to stochastically impute one from another. Future studies are required to explore this issue.

The results of our study highlight the strong correlation between PRO2 and HBI. The simple 2-to-1 estimation between indices facilitates interpretation of clinical trial data and application of guidelines^12^ in clinical practice. Moreover, clinical guidelines emphasize quality of life, which is not captured by PRO2. Harvey-Bradshaw Index provides additional domains of disease to provide some of this information and may be captured in practice. However, the inherent limitations of the current clinical indices emphasize the importance of objective markers in addition to clinical symptoms when making treatment decisions.

## Supplementary Data

Supplementary data is available at *Inflammatory Bowel Diseases* online.

izae281_suppl_Supplementary_Tables_1-7_Figures_1-6
