# Correction to: Estimation of the Harvey Bradshaw Index from the Patient Reported Outcome-2 in Crohn’s Disease

**DOI:** 10.1093/ibd/izaf014

**Published:** 2025-02-11

This is a **correction** to: Reena Khanna, Surim Son, Guangyong Zou, Pavel S Roshanov, Estimation of the Harvey Bradshaw Index from the Patient-Reported Outcome 2 in Crohn’s Disease: Results Based on a Large Scale Randomized Controlled Trial, *Inflammatory Bowel Diseases*, 2024; https://doi.org/10.1093/ibd/izae281

The title of the original publication has been changed. This now reads: “Estimation of the Harvey Bradshaw Index from the Patient Reported Outcome-2 in Crohn’s Disease” instead of: “Estimation of the Harvey Bradshaw Index from the Patient-Reported Outcome 2 in Crohn’s Disease: Results Based on a Large Scale Randomized Controlled Trial”.

Emendation has been made in the article.

