# Supplementary material for: Estimation of the Harvey Bradshaw Index from the Patient-Reported Outcome 2 in Crohn’s Disease: Results Based on a Large Scale Randomized Controlled Trial
Source: Inflamm Bowel Dis. 2024 Dec 4;31(8):2097–105. doi: 10.1093/ibd/izae281 (PMC12342735; doi:10.1093/ibd/izae281)
Supplement: izae281_suppl_Supplementary_Tables_1-7_Figures_1-6 [file izae281_suppl_supplementary_tables_1-7_figures_1-6.docx]

**Supplement**

**Table 1S. Correlation between PRO2 and HBI scores by cohort**

|  | | INDUCTION | | | MAINTENANCE | |
| --- | --- | --- | --- | --- | --- | --- |
|  | Randomized cohort  r (95%CI), p | | Open-label cohort  r (95%CI), p | Randomized cohort  r (95%CI), p | | Open-label cohort  r (95%CI), p |
| Absolute scores | 0.85 (0.82, 0.88), <0.001 | | 0.87 (0.86, 0.89), <0.001 | 0.85 (0.80, 0.90), <0.001 | | 0.88 (0.85, 0.91), <0.001 |
| Change scores | 0.74 (0.69, 0.79), <0.001 | | 0.70 (0.66, 0.74), <0.001 | 0.84 (0.78, 0.89), <0.001 | | 0.80 (0.76, 0.84), <0.001 |

**Table 2S. Linear regression equation for conversion of PRO2 to HBI by cohort**

| INDUCTION | | MAINTENANCE | |
| --- | --- | --- | --- |
| Randomized cohort  (R^2^; p) | Open-label cohort  (R^2^; p) | Randomized cohort  (R^2^; p) | Open-label cohort  (R^2^; p) |
| HBI=0.86+0.46PRO2  (0.72; <0.001) | HBI=0.46+0.48PRO2  (0.76; <0.001) | HBI=0.82+0.42PRO2  (0.73; <0.001) | HBI=0.52+0.46PRO2  (0.78; <0.001) |

**Table 3S. Linear regression equation for conversion of HBI change scores to PRO2 change scores by cohort**

| INDUCTION | | MAINTENANCE | |
| --- | --- | --- | --- |
| Randomized cohort  (R^2^; p) | Open-label cohort  (R^2^; p) | Randomized cohort  (R^2^; p) | Open-label cohort  (R^2^; p) |
| ∆HBI= 0.31+0.45∆PRO2  (0.55; <0.001) | ∆HBI=-0.60 +0.44 ∆PRO2  (0.49; <0.001) | ∆HBI= -0.09+0.45∆PRO2  (0.70; <0.001) | ∆HBI= -0.14+0.44∆PRO2  (0.64; <0.001) |

**Table 4S. Original and optimism-adjusted regression equation**

|  | Original |  | Optimism-adjusted |  |
| --- | --- | --- | --- | --- |
|  | Equation | R^2^ | Equation | R^2^ |
| Week 0 | HBI = 2.2209 + 0.4142PRO2 | 0.5659 | HBI = 2.2213 + 0.4143PRO2 | 0.5648 |
| Week 6 | HBI = 0.5944 + 0.4726PRO2 | 0.7496 | HBI = 0.5946 + 0.4727PRO2 | 0.7489 |
| Week 52 | HBI = 0.6006 + 0.4477PRO2 | 0.7657 | HBI = 0.6005 + 0.4477PRO2 | 0.7634 |
| Week 6 to 0 | ∆HBI = -0.4958 + 0.4469∆PRO2 | 0.5149 | ∆HBI= -0.4969 + 0.4479∆PRO2 | 0.5127 |
| Week 52 to 6 | ∆HBI= -0.1254 + 0.4439∆PRO2 | 0.6618 | ∆HBI = -0.1255 + 0.4443∆PRO2 | 0.6599 |

**Table 5S. Optimism-corrected discrimination and calibration statistics based on 1,000 bootstrap samples**

|  | Optimism-corrected | | | | | Calibration plot | | |
| --- | --- | --- | --- | --- | --- | --- | --- | --- |
|  | R2 | MSE | g-index | Intercept | Slope | Mean absolute error | Mean squared error | 0.9 quantile of absolute error |
| Week 0 | 0.5648 | 6.2275 | 3.1755 | -0.004 | 1.0002 | 0.132 | 0.03635 | 0.282 |
| Week 6 | 0.7489 | 4.9982 | 4.3476 | -0.0015 | 1.0003 | 0.045 | 0.00487 | 0.09 |
| Week 52 | 0.7634 | 3.2334 | 3.5392 | -0.0031 | 0.9999 | 0.109 | 0.01685 | 0.162 |
| Week 6 to 0 | 0.5127 | 9.1846 | 3.4212 | 0.0085 | 1.0023 | 0.159 | 0.06674 | 0.248 |
| Week 52 to 6 | 0.6599 | 6.2065 | 3.7895 | 0.007 | 1.0009 | 0.123 | 0.02145 | 0.229 |

**Table 6S. HBI scores corresponding to PRO2 scores during induction and maintenance by cohort**

|  | INDUCTION | | | | MAINTENANCE | | | |
| --- | --- | --- | --- | --- | --- | --- | --- | --- |
|  | Randomized Population | | Open-Label Population | | Randomized Population | | Open-Label Population | |
| PRO2 | HBI | 95% Prediction Interval | HBI | 95% Prediction Interval | HBI | 95%  Prediction Interval | HBI | 95%  Prediction  Interval |
| 1 | 1.3 | (-3.5,6.2) | 0.9 | (-3.2,5.1) | 1.2 | (-2.2 ,4.7) | 1.0 | (-2.6,4.6) |
| 2 | 1.8 | (-3.1,6.7) | 1.4 | (-2.7,5.6) | 1.7 | (-1.8,5.1) | 1.4 | (-2.2,5.0) |
| 3 | 2.3 | (-2.6,7.1) | 1.9 | (-2.3,6.0) | 2.1 | (-1.4 ,5.5) | 1.9 | (-1.7,5.5) |
| 4 | 2.7 | (-2.1,7.6) | 2.4 | (-1.8,6.5) | 2.5 | (-0.9,6.0) | 2.4 | (-1.3,6.0) |
| 5 | 3.2 | (-1.7,8.0) | 2.9 | (-1.3,7.0) | 2.9 | (-0.5,6.4) | 2.8 | (-0.8,6.4) |
| 6 | 3.7 | (-1.2,8.5) | 3.3 | (-0.8,7.5) | 3.4 | (-0.1,6.8) | 3.3 | (-0.3,6.9) |
| 7 | 4.1 | (-0.7,9.0) | 3.8 | (-0.3,7.9) | 3.8 | (0.4,7.2) | 3.7 | (0.1 ,7.3) |
| 8 | 4.6 | (-0.3,9.4) | 4.3 | (0.1,8.4) | 4.2 | (0.8,7.6) | 4.2 | (0.6,7.8) |
| 9 | 5.0 | (0.2,9.9) | 4.8 | (0.6,8.9) | 4.6 | (1.2,8.1) | 4.6 | (1.0,8.2) |
| 10 | 5.5 | (0.7,10.4) | 5.2 | (1.1,9.4) | 5.1 | (1.6,8.5) | 5.1 | (1.5,8.7) |
| 11 | 6.0 | (1.1,10.8) | 5.7 | (1.6,9.9) | 5.5 | (2.1,8.9) | 5.5 | (1.9,9.1) |
| 12 | 6.4 | (1.6,11.3) | 6.2 | (2.1,10.3) | 5.9 | (2.5,9.3) | 6.0 | (2.4,9.6) |
| 13 | 6.9 | (2.1,11.7) | 6.7 | (2.5,10.8) | 6.3 | (2.9,9.8) | 6.5 | (2.9,10.1) |
| 14 | 7.4 | (2.5,12.2) | 7.1 | (3.0,11.3) | 6.8 | (3.3,10.2) | 6.9 | (3.3,10.5) |
| 15 | 7.8 | (3.0,12.7) | 7.6 | (3.5,11.8) | 7.2 | (3.7,10.6) | 7.4 | (3.8,11.0) |
| 16 | 8.3 | (3.4,13.1) | 8.1 | (4.0,12.2) | 7.6 | (4.2,11.1) | 7.8 | (4.2,11.4) |
| 17 | 8.8 | (3.9,13.6) | 8.6 | (4.4,12.7) | 8.0 | (4.6,11.5) | 8.3 | (4.7,11.9) |
| 18 | 9.2 | (4.4,14.1) | 9.1 | (4.9,13.2) | 8.5 | (5.0,11.9) | 8.7 | (5.1,12.3) |
| 19 | 9.7 | (4.8,14.5) | 9.5 | (5.4,13.7) | 8.9 | (5.4,12.3) | 9.2 | (5.6,12.8) |
| 20 | 10.1 | (5.3,15.0) | 10.0 | (5.9,14.1) | 9.3 | (5.8,12.8) | 9.6 | (6.0,13.3) |
| 21 | 10.6 | (5.8,15.5) | 10.5 | (6.3,14.6) | 9.7 | (6.2,13.2) | 10.1 | (6.5,13.7) |
| 22 | 11.1 | (6.2,15.9) | 11.0 | (6.8,15.1) | 10.1 | (6.7,13.6) | 10.6 | (6.9,14.2) |
| 23 | 11.5 | (6.7,16.4) | 11.4 | (7.3,15.6) | 10.6 | (7.1,14.1) | 11.0 | (7.4,14.6) |
| 24 | 12.0 | (7.2,16.9) | 11.9 | (7.8,16.1) | 11.0 | (7.5,14.5) | 11.5 | (7.8,15.1) |
| 25 | 12.5 | (7.6,17.3) | 12.4 | (8.2,16.5) | 11.4 | (7.9,14.9) | 11.9 | (8.3,15.5) |
| 26 | 12.9 | (8.1,17.8) | 12.9 | (8.7,17.0) | 11.8 | (8.3,15.4) | 12.4 | (8.7,16.0) |
| 27 | 13.4 | (8.5,18.3) | 13.3 | (9.2,17.5) | 12.3 | (8.7,15.8) | 12.8 | (9.2,16.5) |
| 28 | 13.9 | (9.0,18.7) | 13.8 | (9.7,18.0) | 12.7 | (9.1,16.2) | 13.3 | (9.7,16.9) |
| 29 | 14.3 | (9.5,19.2) | 14.3 | (10.1,18.4) | 13.1 | (9.5,16.7) | 13.7 | (10.1,17.4) |
| 30 | 14.8 | (9.9,19.6) | 14.8 | (10.6,18.9) | 13.5 | (10.0,17.1) | 14.2 | (10.6,17.8) |
| 31 | 15.3 | (10.4,20.1) | 15.2 | (11.1,19.4) | 14.0 | (10.4,17.6) | 14.7 | (11.0,18.3) |
| 32 | 15.7 | (10.8,20.6) | 15.7 | (11.6,19.9) | 14.4 | (10.8,18.0) | 15.1 | (11.5,18.8) |
| 33 | 16.2 | (11.3,21.1) | 16.2 | (12.1,20.4) | 14.8 | (11.2,18.4) | 15.6 | (11.9,19.2) |
| 34 | 16.6 | (11.8,21.5) | 16.7 | (12.5,20.8) | 15.2 | (11.6,18.9) | 16.0 | (12.4,19.7) |
| 35 | 17.1 | (12.2,22.0) | 17.2 | (13.0,21.3) | 15.7 | (12.0,19.3) | 16.5 | (12.8,20.1) |
| 36 | 17.6 | (12.7,22.5) | 17.6 | (13.5,21.8) | 16.1 | (12.4,19.7) | 16.9 | (13.3,20.6) |
| 37 | 18.0 | (13.1,22.9) | 18.1 | (13.9,22.3) | 16.5 | (12.8,20.2) | 17.4 | (13.7,21.1) |
| 38 | 18.5 | (13.6,23.4) | 18.6 | (14.4,22.8) | 16.9 | (13.2,20.6) | 17.8 | (14.2,21.5) |
| 39 | 14.8 | (9.9,19.6) | 14.8 | (10.6,18.9) | 13.5 | (10.0,17.1) | 14.2 | (10.6,17.8) |
| 40 | 19.4 | (14.5,24.3) | 19.5 | (15.4,23.7) | 17.8 | (14.0,21.5) | 18.8 | (15.1,22.5) |

Highlighted values represent established thresholds for PRO2.

**Table 7S. HBI change scores corresponding to PRO2 change score in the induction and maintenance phase by cohort**

|  | INDUCTION | | | | MAINTENANCE | | | |
| --- | --- | --- | --- | --- | --- | --- | --- | --- |
|  | Randomized Population | | Open-Label Population | | Randomized Population | | Open-Label Population | |
| PRO2 | HBI | 95% Prediction Interval | HBI | 95% Prediction Interval | HBI | 95% Prediction Interval | HBI | 95% Prediction Interval |
| 1 | -0.8 | (-6.4,4.9) | -1.1 | (-7.2,5.1) | -0.5 | (-5.2,4.2) | -0.6 | (-5.6,4.5) |
| 2 | -1.2 | (-6.9,4.4) | -1.5 | (-7.6,4.6) | -1.0 | (-5.7,3.7) | -1.0 | (-6.1,4.0) |
| 3 | -1.7 | (-7.3,4.0) | -1.9 | (-8.0,4.2) | -1.4 | (-6.1,3.3) | -1.5 | (-6.5,3.6) |
| 4 | -2.1 | (-7.8,3.5) | -2.4 | (-8.5,3.7) | -1.9 | (-6.6,2.8) | -1.9 | (-6.9,3.1) |
| 5 | -2.6 | (-8.2,3.1) | -2.8 | (-8.9,3.3) | -2.3 | (-7.0,2.4) | -2.4 | (-7.4,2.7) |
| 6 | -3.0 | (-8.7,2.6) | -3.3 | (-9.4,2.9) | -2.8 | (-7.5,1.9) | -2.8 | (-7.8,2.2) |
| 7 | -3.5 | (-9.1,2.2) | -3.7 | (-9.8,2.4) | -3.2 | (-7.9,1.5) | -3.2 | (-8.3,1.8) |
| 8 | -3.9 | (-9.6,1.7) | -4.1 | (-10.3,2.0) | -3.7 | (-8.4,1.0) | -3.7 | (-8.7,1.4) |
| 9 | -4.4 | (-10.0,1.3) | -4.6 | (-10.7,1.53) | -4.1 | (-8.8,0.6) | -4.1 | (-9.2,0.9) |
| 10 | -4.8 | (-10.5,0.8) | -5.0 | (-11.1,1.1) | -4.6 | (-9.3,0.1) | -4.6 | (-9.6,0.5) |

Highlighted values represent established thresholds for PRO2.

**Figure S1. Calibration plot with a bootstrap resampling validation for predicting the HBI scores at Week 0**

Ideal line represents a perfect fit.

Apparent line illustrates actual performance with apparent accuracy.

Bias-corrected line displays estimates of the calibration plot adjusted for overfitting.

Apparent and bias-corrected lines were obtained from bootstrap resampling with 1,000 samples for internal validation (mean absolute error = 0.132, mean squared error = 0.03635, 0.9 quantile of absolute error = 0.282).

**Figure 2S. Calibration plot with a bootstrap resampling validation for predicting the HBI scores at Week 6**

Ideal line represents a perfect fit.

Apparent line illustrates actual performance with apparent accuracy.

Bias-corrected line displays estimates of the calibration plot adjusted for overfitting.

Apparent and bias-corrected lines were obtained from bootstrap resampling with 1,000 samples for internal validation (mean absolute error = 0.045, mean squared error = 0.00487, 0.9 quantile of absolute error = 0.09).

**Figure S3. Calibration plot with a bootstrap resampling validation for predicting the HBI scores at Week 52**

Ideal line represents a perfect fit.

Apparent line illustrates actual performance with apparent accuracy.

Bias-corrected line displays estimates of the calibration plot adjusted for overfitting.

Apparent and bias-corrected lines were obtained from bootstrap resampling with 1,000 samples for internal validation (mean absolute error = 0.109, mean squared error = 0.01685, 0.9 quantile of absolute error = 0.162).

**Figure S4. Calibration plot with a bootstrap resampling validation for predicting the HBI change scores from week 0 to week 6**

Ideal line represents a perfect fit.

Apparent line illustrates actual performance with apparent accuracy.

Bias-corrected line displays estimates of the calibration plot adjusted for overfitting.

Apparent and bias-corrected lines were obtained from bootstrap resampling with 1,000 samples for internal validation (mean absolute error = 0.159, mean squared error = 0.0667, 0.9 quantile of absolute error = 0.248).

**Figure S5. Calibration plot with a bootstrap resampling validation for predicting the HBI change scores from week 0 to week 52**

Ideal line represents a perfect fit.

Apparent line illustrates actual performance with apparent accuracy.

Bias-corrected line displays estimates of the calibration plot adjusted for overfitting.

Apparent and bias-corrected lines were obtained from bootstrap resampling with 1,000 samples for internal validation (mean absolute error = 0.045, mean squared error = 0.00487, 0.9 quantile of absolute error = 0.09).

**Figure S6:**

**Induction phase at week 6**


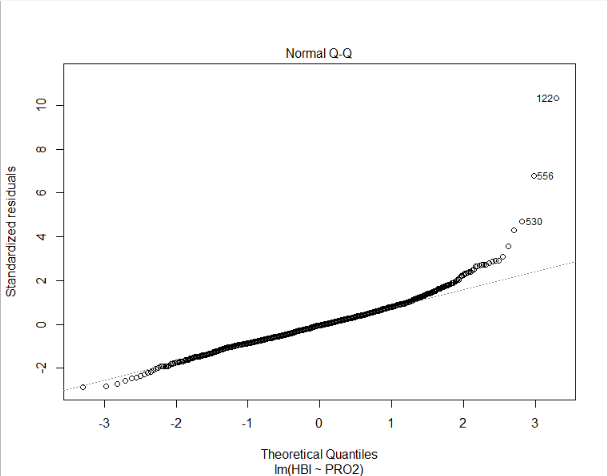

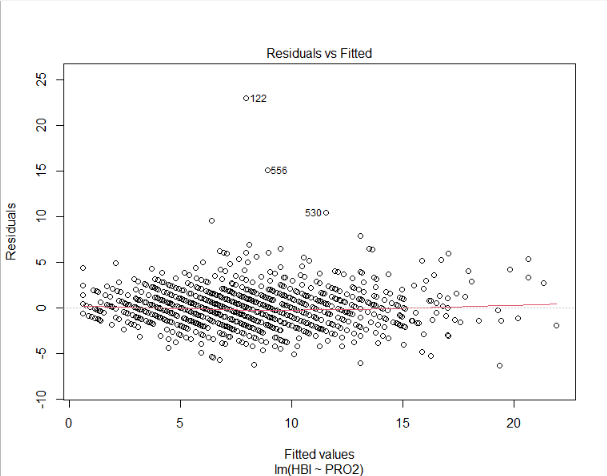


A

B

**Maintenance phase at week 52**


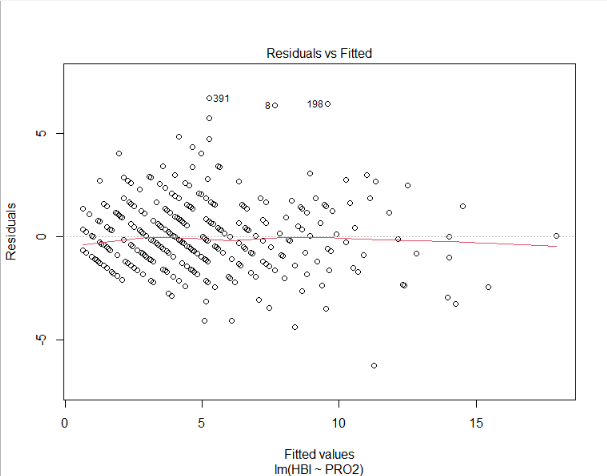

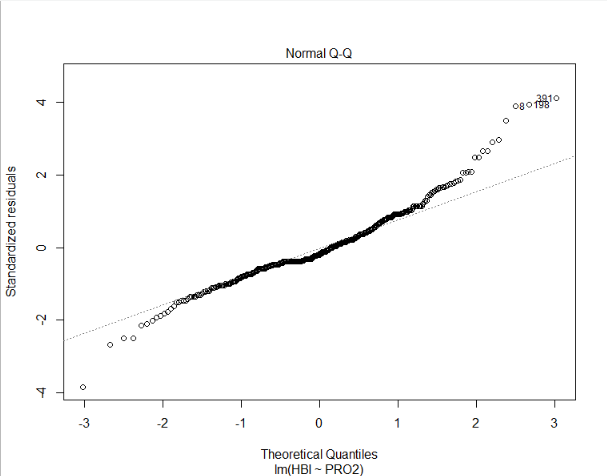


A

B

**Change score from week 0 to week 6 in Induction phase**


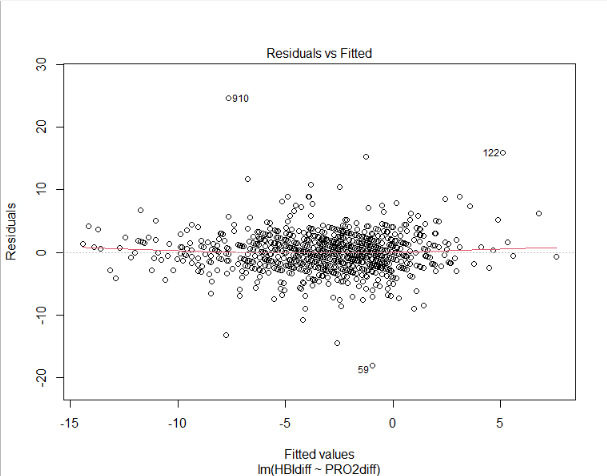

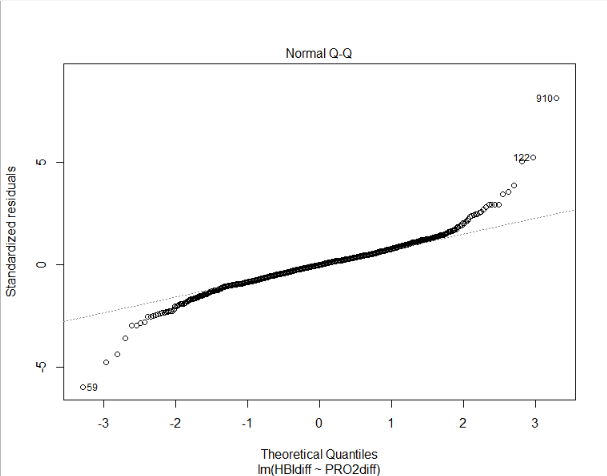


A

B

**Change score from week 6 to week 52 in Maintenance phase**


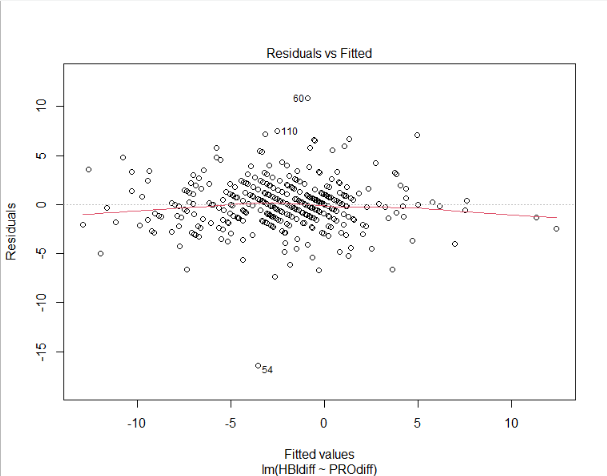

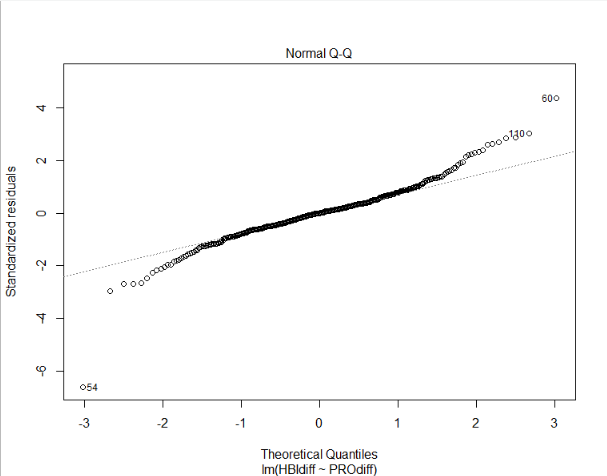


A

B

In all cohorts, the randomly scattered residuals in A) Residual plot suggests that the assumptions of linearity and homoscedasticity are met. In the B) Quantile-Quantile plot, the residuals are closely aligned with the normality line with some deviation observed at both tails. This suggests that the data may have a high peak in the middle and violate the normality assumption, which may affect prediction interval coverage but not the validity of the conversion coefficient estimate.
